# Ancient Out-of-Africa Mitochondrial DNA Variants Associate with Distinct Mitochondrial Gene Expression Patterns

**DOI:** 10.1371/journal.pgen.1006407

**Published:** 2016-11-03

**Authors:** Tal Cohen, Liron Levin, Dan Mishmar

**Affiliations:** Department of Life Sciences, Ben-Gurion University of the Negev, Beer Sheva, Israel; University of Michigan, UNITED STATES

## Abstract

Mitochondrial DNA (mtDNA) variants have been traditionally used as markers to trace ancient population migrations. Although experiments relying on model organisms and cytoplasmic hybrids, as well as disease association studies, have served to underline the functionality of certain mtDNA SNPs, only little is known of the regulatory impact of ancient mtDNA variants, especially in terms of gene expression. By analyzing RNA-seq data of 454 lymphoblast cell lines from the 1000 Genomes Project, we found that mtDNA variants defining the most common African genetic background, the L haplogroup, exhibit a distinct overall mtDNA gene expression pattern, which was independent of mtDNA copy numbers. Secondly, intra-population analysis revealed subtle, yet significant, expression differences in four tRNA genes. Strikingly, the more prominent African mtDNA gene expression pattern best correlated with the expression of nuclear DNA-encoded RNA-binding proteins, and with SNPs within the mitochondrial RNA-binding proteins PTCD1 and MRPS7. Our results thus support the concept of an ancient regulatory transition of mtDNA-encoded genes as humans left Africa to populate the rest of the world.

## Author Summary

The mitochondrion is an organelle found in all cells of our body and plays a significant role in the energy and heat production. This is the only organelle in animal cells harboring its own genome outside of the nucleus. Mitochondrial DNA (mtDNA) variants have been traditionally used as neutral markers to trace ancient population migrations. As a result, the functional impact of human mtDNA population variants on gene regulation is poorly understood. To address this question, we analyzed available data of mtDNA gene expression pattern in a large group of individuals (454) from diverse human populations. Here, we show for the first time that the ancient migration of humans out of Africa correlated with differences in mitochondrial gene expression patterns, and could be explained by the activity of certain RNA-binding proteins. These findings suggest a major mitochondrial regulatory transition, as humans left Africa to populate the rest of the world.

## Introduction

Genetic variants in the nuclear and mitochondrial genomes have been traditionally used to trace the ancient global migration paths of different human populations [1, 2]. Such studies assumed most variants to be neutral and hence merely reflective of the age of the populations studied. However, accumulating evidence suggests that many common variants in the mitochondrial and nuclear genomes have functional impacts [3]. Specifically, ancient mitochondrial DNA (mtDNA) variants and genetic backgrounds (haplotypes, haplogroups) have been associated with an altered tendency to develop a variety of complex traits [3–5] that affected mitochondrial activity in cell culture experiments [6–9] and which conferred adaptive advantages over the course of human evolution [10–12]. Whereas it has been shown that mtDNA variants affected mitochondrial protein activities, such as oxidative phosphorylation (OXPHOS) or the production of reactive oxygen species (ROS), the impact of mtDNA variants on the regulation of gene expression has drawn little attention.

In the nucleus, variants that affect gene expression (eQTLs) can be mapped within intergenic regions, introns and exons [13, 14]. Unlike the nuclear genome (nDNA), most (~93%) of the human mtDNA contains intron-less genes, with the majority of known gene regulatory elements being mapped within the major mtDNA non-coding control region, the D-loop. These transcriptional regulatory elements include the heavy strand and light strand promoters, as well as the three conserved sequence blocks (CSBs I-III). The only known mtDNA gene regulatory element that maps within the coding region is recognized by members of the transcription termination factor family (mTERF) [15]. These proteins correspond to the main regulatory elements that modulate the expression of the entire set of mtDNA genes (N = 37), including those encoding 13 protein subunits of the OXPHOS pathway and 24 RNA components of the mitochondrial translation machinery (22 tRNAs and two rRNAs). All known regulators of mtDNA transcription are imported as proteins from the nucleus [16], namely mitochondrial RNA polymerase (POLRMT), mitochondrial transcription factors A (TFAM) and B2 (TFB2), and mTERF [15]. Recently, however, we and others have shown that additional nDNA-encoded transcription factors, such as MEF2D, the estrogen receptor, c-Jun and Jun-D are imported into mitochondria, where they bind the mtDNA within the coding region outside the D-loop to regulate transcription [17–20]. These findings not only suggest that mtDNA transcriptional regulation is more complex than once thought but also imply that the quest for genetic variants that affect the regulation of mitochondrial gene expression should not be limited to non-coding mtDNA sequences.

The study of eQTLs in the mtDNA lags far behind that of nDNA eQTLs. We were the first to show that an ancient mtDNA control region variants affected *in vitro* transcription and mtDNA copy numbers in cells sharing the same nucleus but differing in their mtDNAs (i.e., cytoplasmic hybrids or cybrids) [21]. Subsequently, two studies that measured mtDNA transcript levels (among other mitochondrial activities) in cybrids revealed differences among certain mtDNA haplogroups [22, 23]. Despite these advances, a worldwide overview of the landscape of mtDNA transcriptional differences in human populations, similar to what is known of nDNA gene expression [24–26], remains lacking, as do mechanistic explanations for the specific observations made. Such an overview is an essential step towards highlighting candidate mtDNA eQTLs.

With this aim in mind, we analyzed RNA-seq data that was recently made available as part of the 1000 Genomes Project for 454 unrelated individuals of Caucasian and sub-Saharan African origin. Our analyses indicated that samples carrying a combination of certain mtDNA variants presented a distinct mtDNA gene expression pattern. Strikingly, the most prominent finding was that all of samples carrying the African mtDNA haplogroup L diverged from the rest in their pattern of expression, suggesting that mtDNA gene expression diverged between people who left Africa and those who remained in the continent, supporting an ancient regulatory difference. Furthermore, the association of such mtDNA gene expression patterns with SNPs within known regulators of mtDNA gene expression shed light on the possible mechanism underlying this phenomenon.

## Results

### Extracting mtDNA-encoded transcripts from human RNA-seq data

**Fig 1:**
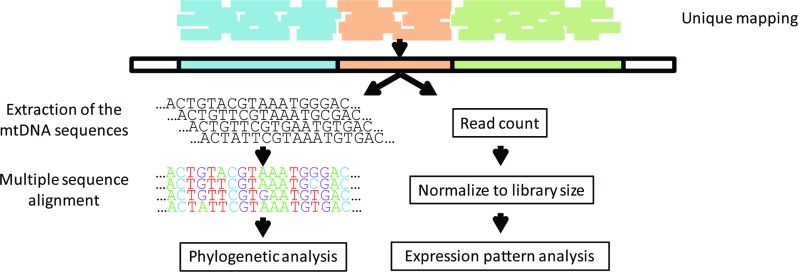
Study design. RNA-seq reads from the Geuvadis consortium were mapped against the human genome, allowing output of reads that were mapped to single loci (unique mapping). The extracted mtDNA sequences were used to (A) perform phylogenetic analysis and (B) as a specific reference sequence for each sample in a remapping process. After remapping, reads were counted and normalized to library size, allowing testing of the expression pattern of each sample in correspondence to its phylogenetic data.

Levels of gene expression can vary among individuals, tissues and species [27]. As such, we utilized RNA-seq experiments to assess differential mitochondrial gene expression patterns among individuals and ethnicities (Fig 1). To this end, we sought RNA-seq studies addressing a variety of human populations. As a first step, we attempted to compile available RNA-seq datasets from various populations [26, 28–31] to generate the largest and most diverse studied cohort. However, expression pattern clustering analysis grouped RNA-seq samples according to the study of origin, even when considering the same samples that were separately sequenced and analyzed independently by different groups (S1 Fig), thus arguing against co-analysis of RNA-seq data generated by different protocols. Hence, although Sudmant et al. [27] recently showed that differences in gene expression patterns between tissues are greater than are differences between studies, our results reveal that while focusing on a single tissue, differences in gene expression patterns between studies exceeds differences among individuals. Therefore, to avoid such artifacts, we focused our analysis on the largest of the relevant studies, encompassing 462 publicly available RNA-seq samples from Caucasians and sub-Saharan Africans [26], all part of the 1000 Genomes Project [32]. This dataset included results from mRNAs and rRNAs sequencing libraries, here referred as the ‘long RNA’ dataset, as well as short-reads sequencing libraries that includes mtDNA-encoded tRNAs (i.e. the tRNA dataset). In that study, all samples were randomly distributed to seven laboratories and RNA-seq data was generated following an identical shared protocol. In considering the 462 RNA-seq samples, eight of the long RNA dataset did not successfully map to human nDNA and mtDNA reference genomes. Our analysis indicated that this problem stems from uneven numbers of paired reads (STAR mapping criterion), which may reflect lower data quality. To avoid possible technical biases we excluded the mentioned 8 samples from further analysis. The number of reads per base that mapped to mtDNA in the remaining 454 long RNA samples ranged from several hundred in the case of tRNA genes, to nearly half a million for some protein-coding genes (S2A Fig). Sequencing reads corresponding to tRNAs were under-represented in the long RNA dataset likely due to the library preparation protocols used, which involved a size selection step. We partially overcame this limitation by analyzing the tRNA dataset. Here, 16 of the 22 tRNA genes were represented in the tRNA dataset with sufficient numbers of mapped reads for analysis in at least 90% of the samples. For the sake of consistency, we included only those individuals who were represented in the long RNA dataset when considering the tRNA dataset, thereby retaining 440 samples with coverage of up to tens of thousands mapped reads per mtDNA base (S2B Fig).

Lappalainen et al. [26] reported that there were no significant differences between the same samples that were generated in different laboratories. This enabled us to divide the entire dataset into two groups matched according to ethnicity and gender ratio, which were separately treated as biological replicates.

Recently, we, and others, identified RNA-DNA differences (RDD) in three mtDNA sites common to all human individuals and human tissues tested to date [33, 34], as well as in 90% of the vertebrates [35]. Our sequence analysis of the long RNA dataset also identified these three RDDs in all analyzed samples, while excluding sequencing and mapping errors by analysis filters, as previously outlined [33], thus further supporting the quality of our analyzed data.

### Using RNA-seq-based mtDNA sequences to reconstruct known mtDNA phylogenetic tree topology

The polycistronic nature of mtDNA transcription permitted RNA-seq reads that covered the complete mtDNA of all tested individuals. This enabled reconstruction of the entire mtDNA sequence in all analyzed samples, which were aligned to reveal polymorphic positions and reconstruct a phylogenetic tree (S3A Fig). Such analysis enabled the assignment of all individuals to specific mtDNA haplogroups. Since the analyzed samples are part of the 1000 Genomes Project, we extracted the mtDNA sequence from the DNA sequence database of the same samples and used these to construct a phylogenetic tree (S3B Fig). Notably, the topologies and distribution of haplogroups throughout the RNA and DNA-based trees were nearly identical and were in agreement with previously published trees [12, 36]. Therefore, putative human RNA-DNA sequence differences did not affect overall mtDNA tree topology.

### Mitochondrial nDNA pseudogenes (NUMTs) likely did not impact expression differences

We and others previously showed that nDNA harbors a repertoire of mtDNA sequence fragments (NUMTs) that were transferred from the mitochondria during the course of evolution [37, 38]. NUMTs potentially pose an obstacle to mtDNA gene expression assessment, as a subset of RNA reads might originate from NUMTs rather than from the active mtDNA. As a first step to control for such a scenario, we remapped the RNA-seq reads of each sample against their own reconstructed mtDNA sequence (personalized mapping). This approach also controlled for a second possible bias. It is conceivable that gene expression level differences could be affected by exclusion of sequencing reads due to mapping of the RNA-seq reads to a single European reference sequence (the revised Cambridge Reference Sequence (rCRS), i.e. rCRS mapping), resulting in the exclusion of numerous variants [39]. Since the mtDNA is highly variable and since ~130,000 years separates the appearance of the L haplogroup from the remaining mtDNA genetic backgrounds analyzed [10, 40], our sample-specific analysis enforced increasingly accurate and unique read mapping while excluding erroneous mapping to more than a single locus. Secondly, paired-end technology enabled us to exclude reads whose paired read partner mapped to a non-mtDNA locus. Finally, we repeated our read mapping while avoiding the unique-mapping step and compared the results obtained to those realized by unique-mapping analysis. The latter analysis did not reveal any skew in the expression pattern observed for those samples analyzed. We, therefore, concluded that NUMTs had little or no impact on our data.

### The African L-haplogroup shows lower expression of mtDNA-encoded genes than non-Africans

**Fig 2:**
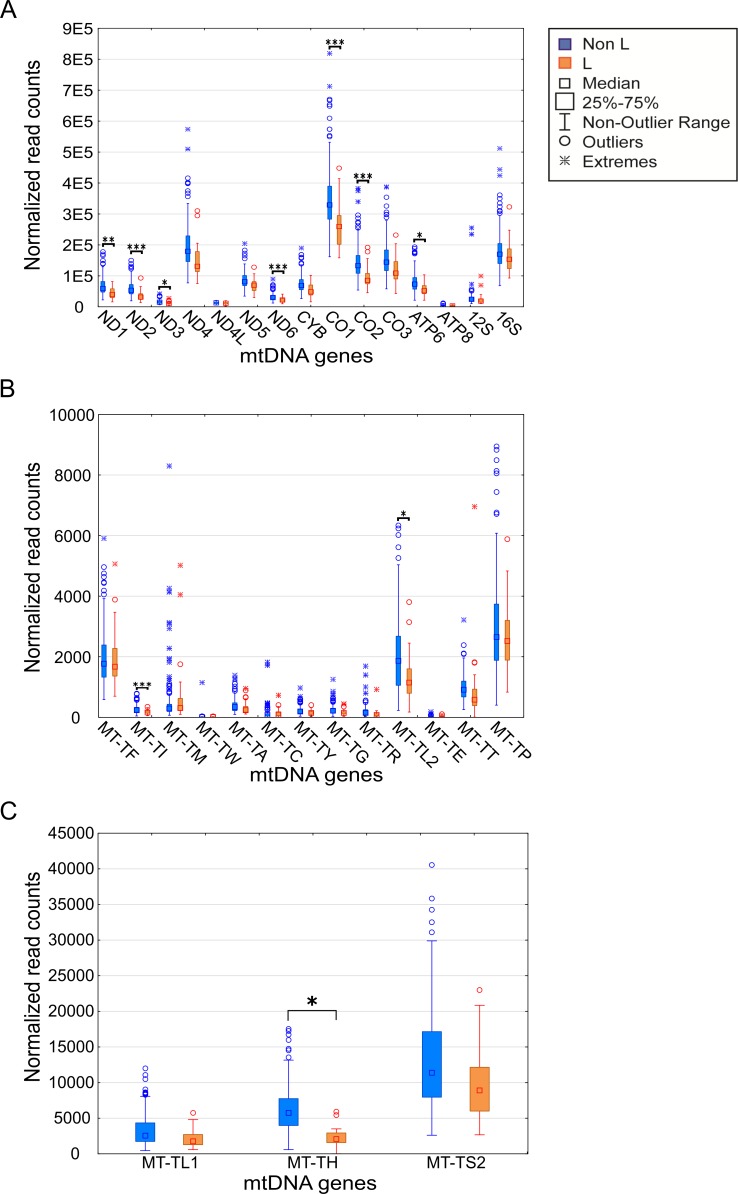
mtDNA gene expression is lower in L-haplogroup individuals. Expression levels of the mtDNA genes in L-haplogroup and non-L-haplogroup individuals. Long RNA dataset (protein-coding genes and rRNA) (A). X axis–mtDNA genes, Y axis–DESeq normalized read count. (B) and (C) represent the tRNA dataset with lower and higher expression levels, respectively. Axes are as described in (A). Statistical significance: (*) p<3.7e-5; (**) p< 1e-6; (***) p< 1e-7.

We asked whether certain mtDNA SNPs associate with differential expression levels of mtDNA-encoded genes. Since we analyzed multiple mtDNA SNPs (including both singletons and lineage-defining SNPs), Bonferroni correction for multiple testing was applied. As mentioned above, initial analysis was performed while randomly dividing the samples into two groups while retaining the proportions of gender and ethnicity. Such analysis, using the personalized mapped samples, revealed correlation between certain SNPs and a distinct expression pattern. Close inspection revealed that all these SNPs corresponded to mtDNA haplogroup L (Fig 2, S1 Table, S2 Table). It is worth noting that analysis based on either the personalized- or rCRS-mapped samples led to comparable expression patterns (S4 Fig). This was despite the fact that the personalized mapping exhibited with excess of mapped reads in L halogroup samples, i.e. a mean of additional 26,197 reads per sample–a 0.09% increase, in the personalized mapping samples. Similarly, there was a slight increase in the number of reads in personalized mapped Caucasian samples, i.e. a mean of additional 5,279 reads per sample–a 0.02% increase. Taken together, regardless of the mapping approach, we conclude that L haplogroup individuals displayed reduced levels of mtDNA gene expression. For the sake of simplicity further analyses were performed using the personalized mapped samples.

**Fig 3:**
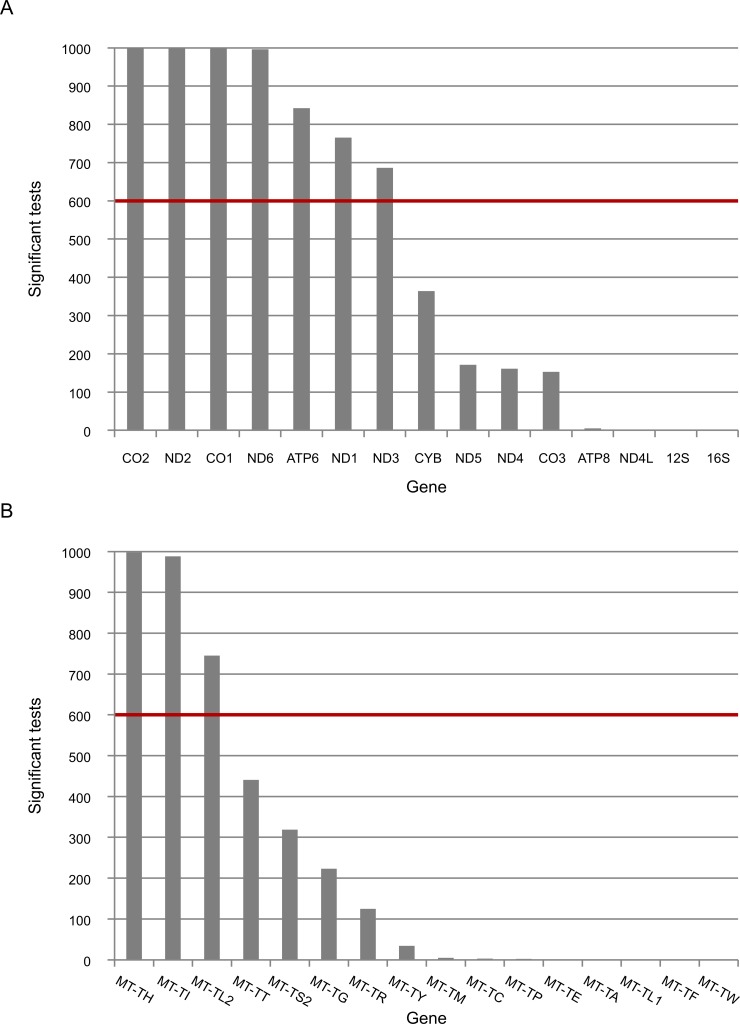
The expression of five mtDNA genes that diverged between L- and non-L-haplogroups in replicated association analyses. Presented is the number of times that the expression of mtDNA genes was associated with L-haplogroup SNPs for the long RNA (A) and tRNA (B) datasets. The red line represents the cutoff value (60%). X axis–mtDNA annotation for protein (A) or tRNA genes (B), Y axis–absolute number of replicated associated analyses of mtDNA SNPs and mtDNA gene expression.

To control for possible bias underlying the trend towards lower levels of L-haplogroup mtDNA transcript expression, we considered the expression patterns of nDNA-encoded genes in Africans versus non-Africans. We found 2,380 nDNA-encoded genes that are differentially expressed in Africans (S3 Table), yet unlike the mtDNA genes ~54% showed higher expression, while the rest showed lower expression in the African group (S5 Fig). These findings suggest a lack of bias in the expression pattern of mtDNA-encoded transcripts. To control for possible group assignment bias, we randomly re-divided the samples 500 times, while retaining constant proportions of gender and ethnicities. Following group assignment, we repeated the gene expression normalization process and SNP association analysis. Our results revealed that in more than 60% of the replicated divisions, ten mtDNA-encoded genes (MT-TH, MT-TI, M-TL2, MT-CO2, MT-ND2, MT-ND6, MT-CO1, MT-ATP6, MT-ND3 and MT-ND1) consistently showed significantly reduced expression levels in L-haplogroup samples (Fig 3). These results confirm that African L-haplogroup individuals possess a distinct mtDNA gene expression pattern.

**Fig 4:**
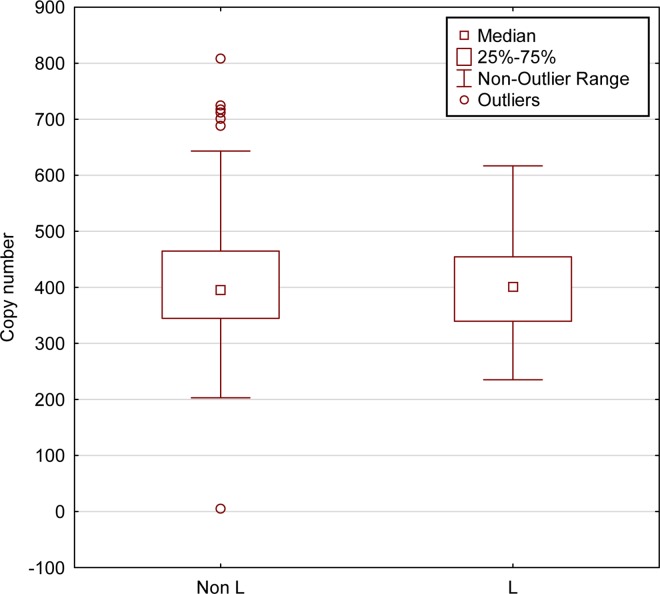
mtDNA copy numbers do not differ between L-haplogroup and non-L-haplogroup individuals. mtDNA copy numbers were calculated for individuals for which DNA-seq was available (N = 436, 1000 Genomes Project). No differences were detected in mtDNA copy numbers between L-haplogroup and non-L-haplogroups (Kruskal-Wallis test, KW-H (1436) = 0.262, p = 0.6088). X-axis–L-haplogroup versus non-L-haplogroup individuals, Y-axis–calculated mtDNA copy numbers.

Since mtDNA transcription and replication are coupled in human mitochondria [41], we included mtDNA copy number as one of the covariates in all our eQTL analyses. Nevertheless, we tested whether the differences in expression levels associated with variations in mtDNA copy numbers. We found that variations in mtDNA copy numbers did not differ between L- and non L-haplogroup mtDNAs (Fig 4). This suggests that the variation we observed in mtDNA gene expression patterns was independent of mtDNA copy numbers, a finding in agreement with previous results [42].

### Specific mtDNA haplogroups exhibit with distinct gene expression patterns

**Fig 5:**
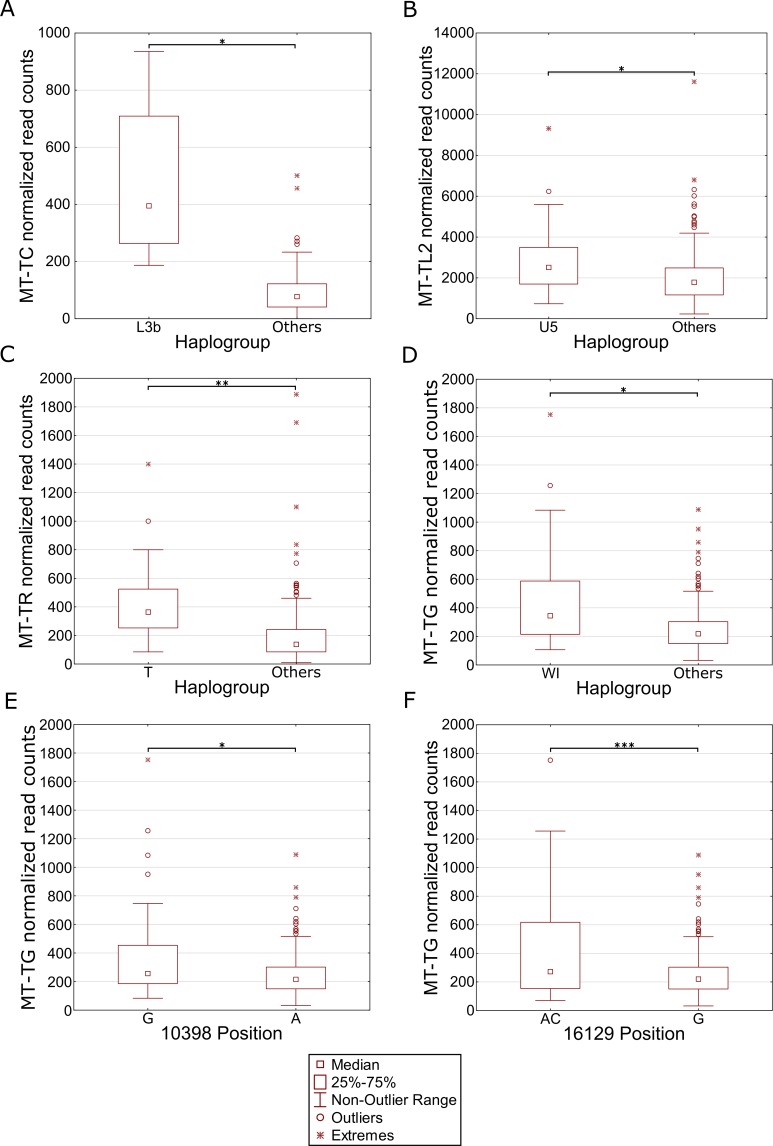
Differential expression of mtDNA genes in different haplogroups within populations. Each panel represents a gene that differentially expressed within individuals harboring certain common SNPs in the African (A) and Caucasian (B-F) populations. X axis—SNP-associated haplogroups; 10,398 and 16,129 –mtDNA positions with common recurrent SNPs that showed differential expression; Y axis- normalized read counts. Statistical significance: (*) p< 3.55e-5; (**) p< 1e-6; (***) p< 1e-7.

**Fig 6:**
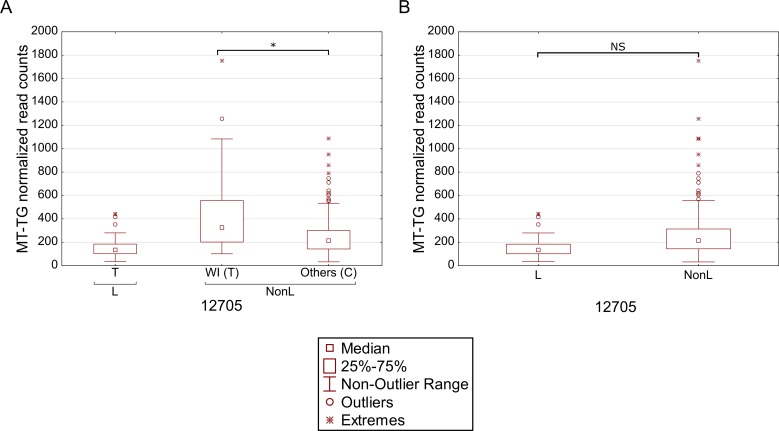
Masking of mtDNA expression differences within populations. Differential expression of tRNA glycine in individuals with either the T or C alleles in mtDNA position 12,705, considering Caucasians and Africans, separately (A). Comparison of tRNA glycine expression in the entire dataset (B). L- Africans; NonL- Caucasians. X axis—SNPs within each haplogroup; Y axis- normalized read counts.

We reasoned that the highly significant gene expression differences between Africans and Caucasians may mask intra-population expression variation. To address this possibility we repeated the gene expression analysis separately for Caucasians and Africans. Although this analysis did not reveal any significant intra-population differences while considering the long RNA dataset (S4 Table, S5 Table), our results indicate that in Africans, individuals belonging to haplogroup L3b had significantly higher expression of cysteine tRNA (Fig 5A and S6 Table). While analyzing the Caucasian samples (Fig 5B–5F and S7 Table), we found that tRNA Leucine (2) had higher expression in individuals belonging to haplogroup U5. Secondly, higher expression of tRNA arginine was found in individuals belonging to haplogroup T. Finally, tRNA glycine had higher expression level in individuals sharing SNPs that define haplogroup cluster WI, in individuals harboring a guanine as compared to those having an adenine allele in mtDNA position 10,398 (shared by haplogroups J, K and I), and in individuals with either an adenine or a cytosine in mtDNA position 16,129 as compared to those with a guanine in this position. Hence, our intra-population analysis revealed much significant variation in mtDNA gene expression that was previously masked by the more prominent differential expression between Africans and Caucasians. Such differences may stem, at least in part, from variation in the impact of certain alleles on gene expression, depending on their linked haplotypes (Fig 6). This is best exemplified by the relatively high expression of tRNA glycine in Caucasian haplogroup cluster WI individuals (with the 12,705T allele) as compared to individuals with the 12,705C allele (see also Fig 5D); all Africans harbor the 12,705T allele, which exhibits even lower tRNA glycine expression than the Caucasian 12,705C allele. The latter caused lack of significance while calculating the impact of 12,705 SNPs on gene expression considering Africans and Caucasians together (Fig 6B). Taken together, the impact of mtDNA SNPs on gene expression differences is modified, at least in part, by their linked genetic background.

### RNA-binding genes are co-expressed with L-haplogroup mtDNA genes

Since the regulation of all mitochondrial activities is governed by nDNA-encoded factors, we asked which nDNA-encoded genes are the best candidates to modulate the most prominent distinct mtDNA gene expression pattern–that of the L-haplogroup. As a first step in addressing this question, we screened for nDNA-encoded genes that were co-expressed with the mtDNA genes (Pearson correlation). These genes were then subjected to Matrix eQTL [43] analysis to identify differential expression between mtDNA genetic backgrounds (i.e., L or non-L haplogroups), while including gender, mtDNA copy number, and sample resource lab as covariates. After further correction for multiple testing (Bonferroni correction), a list of 2,380 genes remained (S3 Table). GO analysis revealed enrichment in RNA-binding proteins and poly-adenylated RNA-binding proteins (Table 1, S8 Table), suggesting that RNA stability likely plays a significant role in the differential expression of mitochondrial genes in L-haplogroup individuals.

**Table 1:** GO-term analysis of nDNA-encoded genes that co-expressed with mtDNA genes in L-haplogroup individuals. Shown are the five most significant results

GO-term	Observed	Expected	Fold Enrichment	P-value
RNA binding	368	150.2	2.45	2.30e^-53^
nucleic acid binding	659	375.17	1.76	1.38e^-48^
poly(A) RNA binding	289	107.65	2.68	6.24e^-48^
heterocyclic compound binding	854	548.95	1.56	8.49e^-46^
organic cyclic compound binding	859	556.48	1.54	9.03e^-45^

### SNPs in mitochondrial RNA-binding proteins are associated with the L-haplogroup gene expression pattern

**Fig 7:**
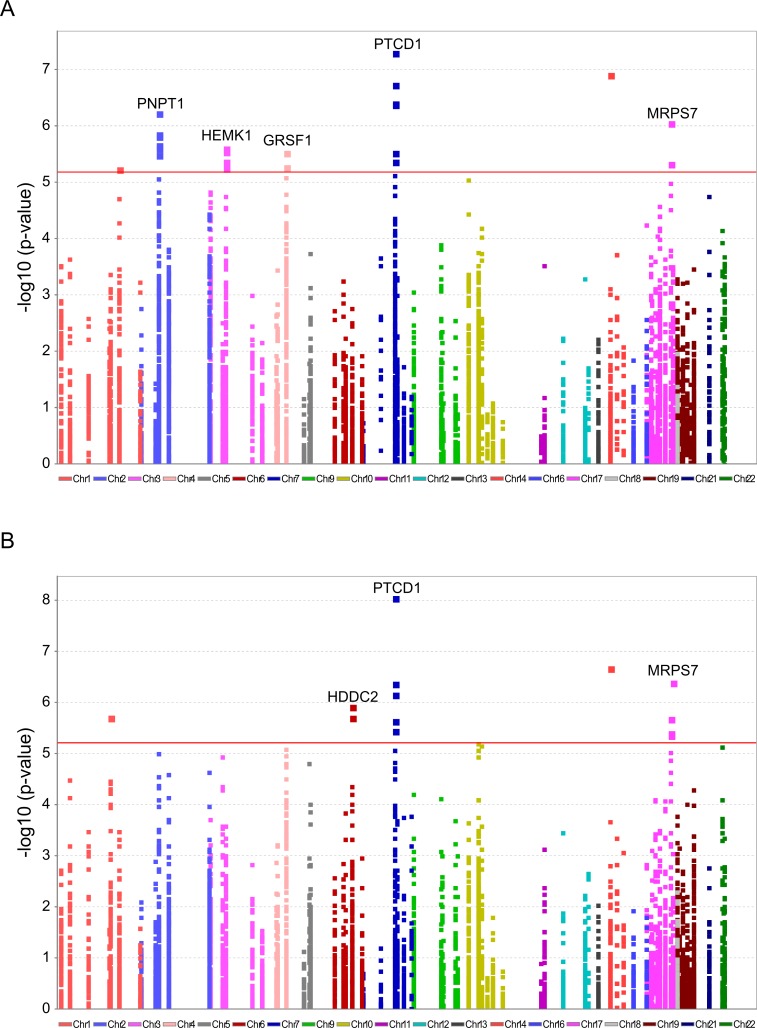
Significant association of nDNA SNPs with African-Caucasian differences in mtDNA gene expression. Annotated are nDNA genes that were repeatedly identified in the long RNA (A) and the tRNA (B) datasets. X axis–SNP distribution across human chromosomes, Y axis–logP values. P-value of significance (after Bonferroni correction = 6.52e^-6^ for the long RNAs and 6.11e^-6^ for the tRNAs) is indicated by a horizontal line.

To further explore candidate genes that best explain the distinct L-haplogroup mtDNA expression pattern, we screened for nDNA SNP association. To avoid statistical power issues, we focused our SNP association study on the most comprehensive list of human RNA-binding and transcription-associated mitochondrial genes available [16], supplemented by additional transcription factors that were recently localized to the human mitochondria and bind the mtDNA [18, 19]. This analysis showed 7,665 correlations between a total of 511 non-redundant nDNA SNPs and 15 mtDNA-encoded genes, of which only five SNPs in four nDNA-encoded genes remained significant after correction for multiple testing in both test groups. Notably, these SNPs correlated with the expression of four mtDNA-encoded genes, namely MT-ND2, MT-CO2, MT-CO1 and MT-ND6 (Fig 7A, Table 2, S9 Table).

**Table 2:** nDNA SNPs that associate with the African-Caucasian differences in mtDNA expression from the long RNA dataset.

**Chromosome**	**SNP accession number**	**nDNA Gene**	**SNP location in the nDNA gene**	**mtDNA gene with altered expression**	*p-value
7	rs28495024	PTCD1	UTR-3	ND2	4.84e^-8^
7	rs28495024	PTCD1	UTR-3	CO2	3.86e^-7^
7	rs2003499	PTCD1	UTR-3	CO2	2.89e^-6^
17	rs16967742	MRPS7	UTR-5	ND2	8.38e^-7^
17	rs16967742	MRPS7	UTR-5	CO2	4.36e^-6^
2	rs6739788	PNPT1	UTR-3	CO2	2.11e^-6^
2	rs7594497	PNPT1	Exon N>D	CO1	2.41e^-6^
2	rs7594497	PNPT1	Exon N>D	CO2	3.14e^-6^
3	rs667894	HEMK1	UTR-3	ND2	2.38e^-6^
3	rs667894	HEMK1	UTR-3	CO1	4.02e^-6^
3	rs667894	HEMK1	UTR-3	CO2	2.71e^-6^
3	rs667894	HEMK1	UTR-3	ND6	5.27e^-6^
4	rs10938024	GRSF1	UTR-3	ND2	2.90e^-6^
4	rs7694924	GRSF1	UTR-3	ND2	5.12e^-6^

*P-values were calculated using the Matrix eQTL R-package.

The indicated SNPs map within the nDNA-encoded genes PTCD1, MRPS7, PNPT1, HEMK1 and GRSF1. Three of these nDNA genes have already been analyzed for their involvement in mitochondrial gene expression. PNPT1 is involved in RNA import into the mitochondria and is a part of the mitochondrial RNA degradosome [44, 45]. PTCD1 takes part in processing primary mtDNA polycistronic transcripts [46] and GRSF1 is involved in processing both the full and tRNA-less polycistrons [47]. The fourth gene, MRPS7, is a mitochondrial ribosomal protein, and therefore takes part in the mitochondrial translation machinery [48] and HEMK1 methylates the mitochondrial translation release factor HMRF1L [49]. In conducting the same analysis on the tRNA dataset, two of the identified SNPs correlated with the differential expression of the L-haplogroup in both groups of RNA-seq samples, namely PTCD1 and MRPS7 (Fig 7B, Table 3, S10 Table). In summary, our eQTL association analysis of reduced mtDNA gene expression in L-haplogroup individuals revealed consistent association of SNPs within PTCD1 and MRPS7, both in the large and the tRNA datasets, suggesting a common mechanism involved.

**Table 3:** nDNA SNPs that associate with the African-Caucasian differences in mtDNA expression in the tRNA dataset.

Chromosome	SNP accession number	Gene	SNP location in the gene	mtDNA gene with altered expression	*p-value
7	rs28495024	PTCD1	UTR-3	MT-TI	8.59e^-9^
7	rs2003499	PTCD1	UTR-3	MT-TI	3.96e^-7^
17	rs16967742	MRPS7	UTR-5	MT-TI	1.93e^-6^

*P-values were calculated by Matrix eQTL.

## Discussion

In this study, while examining RNA-seq data from 454 individuals of African and Caucasian origin from the 1000 Genomes Project, we found distinct patterns of transcript abundance in individuals with certain mtDNA SNPs, corresponding to the mtDNA L-haplogroup (the most abundant African haplogroup). L-haplogroup individuals presented an overall lower expression of certain mtDNA genes (both mRNAs and tRNAs), as compared to individuals corresponding to non-L-haplogroups. Such distinct mtDNA gene expression patterns of the L-haplogroup suggest an ancient regulatory trait that likely existed prior to out-of-Africa migrations. In the future, when large Asian RNA-seq data become available, it will be possible to corroborate this interpretation.

Our mtDNA eQTL analysis, which revealed a significant expression pattern difference between Africans and Caucasians, was based on SNP-expression pattern association, and was not based on prior division into populations. Furthermore, while performing intra-population eQTL analysis we found distinct mtDNA gene expression pattern for specific haplogroups, only while considering the tRNA genes. Finally, we noticed that the expression of tRNA glycine was elevated in individuals belonging to haplogroups W and I, as well as in individuals with a guanine allele in mtDNA position 10,398 and in individuals with either an adenine or a cytosine in position 16,129 (which is found in people belonging to multiple haplogroups). Interestingly, all haplogroup I individuals harbor a 10,398G allele, suggesting that haplogroup I SNPs play a major role in determining differential expression of tRNA glycine. Alternatively, since the SNPs in positions 10,398 and 16,129 occurred multiple independent times during the evolution of man, it is possible that these SNPs have direct functional impact on the expression of tRNA glycine. We interpret all these results to mean that the intra-population expression differences might be context-dependent, and therefore, they were masked by including both Africans and Caucasians in the same analysis. Specifically, masking of such expression differences may occur, at least in part, due to differential impact of certain SNPs on expression, depending on their linked genetic backgrounds (Fig 6). Such differential impact could further be explained by: (A) the eQTL has a small functional impact which is enhanced by additional eQTLs, or (B) the causal eQTL is in linkage with the identified eQTL. However, currently we cannot differentiate which of the suggested explanations is the most plausible. Taken together, our eQTL analysis was not confounded by populations, and therefore revealed candidate mtDNA-encoded eQTLs.

A recent study of mitochondrial activity in six cell lines sharing the same nDNA but diverging in their mtDNAs (i.e., cybrids), revealed differences in activity and transcript abundance among three L-haplogroup and three H-haplogroup cybrids [23]. Similarly, Gomez-Duran and colleagues identified expression pattern differences between haplogroup H cybrids when compared with those of the haplogroup Uk, 5 cell lines each [22]. Since we studied a much larger sample size from highly diverse individuals, we argue that our study better represents the natural population rather than focusing on specific haplogroups. This further underlines the future need to expand our study to include Asians so as to shed further light on mitochondrial regulatory differences from a world-wide perspective. Once cybrid technology has been adapted for high throughput analysis, it would be of interest to apply our genomic analysis to a large collection of cybrids with diverse mitochondrial genomes.

Since the distinct L-haplogroup mtDNA expression pattern was shared between tRNAs and long RNAs that are encoded by both mtDNA strands, it is plausible that the observed differences stem either from early stage transcription or from polycistron stability. Alternatively, since expression pattern differences were limited to certain mtDNA-encoded genes, the underlying mechanism could involve differences in the RNA stability of the mature transcripts or during transcript maturation, as previously suggested [50]. With this in mind, both analysis of co-expressed nDNA-encoded genes and our eQTL association study revealed that RNA-binding proteins with mitochondrial function (i.e., PTCD1 and MRPS7) best explain the distinct mtDNA gene expression patterns of L-haplogroup individuals. Although a lack of association with SNPs in the vicinity of known mtDNA transcription regulators was observed, one cannot exclude future detection of such association when more mtDNA transcription regulators are identified.

In summary, the distinct mtDNA transcript expression pattern observed in African individuals supports an ancient mitochondrial phenotype, as humans left Africa to populate the rest of the world. We identified several candidate nDNA-encoded modulators of this expression pattern, although their direct functional impact remains to be studied. Nevertheless, expression differences were only seen in certain mtDNA genes, despite the fact that all mtDNA genes are co-transcribed in two polycistrons corresponding to the light and heavy strands. This finding, along with the observed association of SNPs in mitochondrial RNA-binding genes, suggests that RNA decay is the best candidate mechanism for modulating the observed expression pattern.

## Materials and Methods

### Available samples for analysis

RNA-seq data from lymphoblastoid cell lines (LCL) were obtained from several inter-population studies [26, 28–31]. Datasets were downloaded from:

citation 28 - www.ncbi.nlm.nih.gov/geo/query/acc.cgi?acc=GSE19480;

citation 29 - www.ebi.ac.uk/arrayexpress/experiments/E-MTAB-197/;

citation 26 - www.ebi.ac.uk/ena/data/view/ERR188021-ERR188482 and www.ebi.ac.uk/ena/data/view/ERR187488-ERR187939;

citation 30 - www.ncbi.nlm.nih.gov/geo/query/acc.cgi?acc=GSE54308;

citation 31 - www.ncbi.nlm.nih.gov/sra/?term=SRP026597.

However, since transcript abundance assessment (see below and in the Results section) revealed mtDNA gene expression clusters according to these studies, our analysis focused on data in the largest recently published study so as to avoid analysis artifacts [26]. In that study, RNA-seq libraries were constructed and data were generated in seven different labs that followed the same experimental protocol, relying on randomly distributed samples. This dataset included results from sequenced mRNA and rRNA (‘long RNA’) libraries, as well as small RNA libraries. The largest RNA dataset contained samples from 462 individuals from five world-wide populations (91 CEU, 95 FIN, 94 GBR, 93 TSI and 89 YRI). Eight of the samples were not successfully mapped to the human genome due to too much unpaired reads and were, therefore, excluded, thus leaving 454 samples for further analysis. Small RNA sequences generated from a subset of these samples (N = 452) encompassed most of the individuals described above (87 CEU, 93 FIN, 94 GBR, 89 TSI and 89 YRI). Three of the samples were not included in the long RNA dataset and were, therefore, excluded from further analysis, in addition to eight samples whose RNA sequences could not be mapped to the long RNA dataset. The same was true for an additional sample that was not successfully mapped to the dataset of small RNA libraries (tRNAs in the mtDNA). This left 440 samples for further analysis. Data were downloaded from Arrayexpress (E-GEUV-1 and E-GEUV-2 for long RNA and tRNA, respectively).

The long RNA dataset was downloaded from: http://www.ebi.ac.uk/ena/data/view/ERR188021-ERR188482

The small RNA sequence libraries were downloaded from: http://www.ebi.ac.uk/ena/data/view/ERR187488-ERR187939

### Reconstruction of complete mtDNA sequences from RNA-seq data, multiple sequence alignment and phylogenetic analysis

Taking advantage of the polycistronic transcription of the mitochondrial genome [51], mtDNA genomes were reconstructed from each of the RNA-seq samples using MitoBamAnnotator [52]. Each reconstructed mtDNA sequence underwent haplogroup assignment using HaploGrep [53]. Finally, multiple sequence alignment and phylogenetic analysis (neighbor joining, 1000 X bootstrap and default parameters) of all reconstructed mtDNA genomes were performed using MEGA 5 [54].

### Mapping RNA-seq reads to human mtDNA

RNA-seq reads of the long RNA dataset were mapped onto the entire human genome reference sequence (GRCh 37.75) using STAR v2.3 [55] and the available genome files at the STAR website (code.google.com/p/rna-star/). Mapping was performed using default parameters, in addition to the [—outFilterMultimapNmax 1] parameter to achieve unique mapping. Since mtDNA sequence variability can impact the number of mapped RNA-seq reads, the reads were remapped against the same human genome files after replacing the mitochondrial reference sequence by the reconstructed mtDNA of each of the relevant analyzed individuals. To this end, a revised index was generated for the new reference genome by replacing the human mtDNA sequence with the reconstructed version. This was conducted separately for each tested sample, with all other files being retained. Most of the parameters used were retained, with one exception. Apart from replacing the mtDNA reference, we further increased our mapping accuracy by allowing fewer mismatches [—outFilterMismatchNmax 8], while analyzing couples of paired reads (a total length of 150bp). The tRNA dataset was mapped using the same parameters and references as in the remapping process described above, with the single exception of no mismatches allowed [—outFilterMismatchNmax 0] so as to reduce mapping errors [56].

### Estimation of transcript abundance

Alignment files (SAM format) were compressed to their binary form (BAM format) using Samtools [57] with the default parameter [view -hSb] selected, and sorted using the [sort] parameter. Mapped reads were counted using HTSeq-count v0.6.1.p1 [58], employing the [-f bam -r pos -s no] parameters. Reads were normalized to library size using DESeq v1.14.0 [59] and the default parameters. This protocol was employed for both the long RNA and tRNA datasets.

### Expression pattern analysis considering mtDNA SNPs

mtDNA sequences of all individuals were aligned to identify polymorphic positions. In the tRNA dataset, some tRNA genes had no reads in a subset of our analyzed samples. Therefore, only genes presenting with reads in more than 90% of the samples were used, thus leaving 16 tRNA genes for further analysis. For each polymorphic position, the samples were divided into groups according to their allele assignment. As described in Lappalainen [26] et al., using the linear model implemented in the Matrix eQTL R package [43], eQTL mapping was calculated according to the allele assignment, while considering gender, mtDNA copy number and sample resource (i.e. lab of origin) as covariates. A Bonferroni correction was employed to correct for multiple testing. To reduce false positive discovery rate we focused on SNPs shared by at least 10 individuals. To identify possible associations of nDNA-encoded genes with differential expression patterns of mtDNA genes, the analysis focused on known SNPs (as listed by the 1000 Genomes Project) in the dataset of genes with known mitochondrial RNA-binding activity [16]. This dataset was supplemented by transcription factors and RNA-binding proteins that were recently identified in human mitochondria but were not included in MitoCarta (i.e., c-Jun, JunD, CEBPb, Mef2D) [18, 19]. Such prioritization was employed to enable detection of possible correlations with sufficient statistical power. Since our analysis of the mtDNA revealed sites with more than 2 alleles (i.e. 3 alleles at the most) we performed our analysis such that the major allele frequency will not exceed 95%, thus enabling the discovery of the two other minor alleles.

### Identification of nDNA-encoded genes that co-expressed with mtDNA-encoded genes, and differ between populations

To assess co-expression of nDNA-encoded genes with the mtDNA-encoded ones, Pearson correlation was employed (p < 5.23e-8, after Bonferroni-correction for multiple testing). Briefly, co-expression was sought between the 15 mtDNA-encoded mRNA and rRNA genes and 63,662 nDNA-encoded genes. Then, Matrix eQTL [43] was employed to identify differential expression between L and non-L genetic backgrounds among the significantly co-expressed genes, while including gender, mtDNA copy number, and sample resource lab as covariates. Finally, only nDNA-encoded genes that were significantly co-expressed, and were differently expressed between African and Caucasians (p < 5.5e-6, Bonferroni corrected for multiple testing), were subjected to gene ontology (GO) analysis. To this end, PANTHER [60] was used, categorized according to molecular function.

### Estimation of mtDNA copy numbers

As the cell lines that were used in the analyzed RNA-seq study were derived from individuals included in the 1000 Genomes Project [61], mapped DNA reads files were downloaded from the 1000 Genomes Project ftp website in BAM format (ftp://ftp.1000genomes.ebi.ac.uk/vol1/ftp/data), using Samtools with the [view -hb] parameter. Bedtools was employed to estimate the read coverage of nDNA regions in each individual, using the BAM and global BED files of that individual. Using these files, read coverage over most of the mtDNA sequence (mtDNA positions 1–16,499) was compared to that of randomly selected sets of 100,000 bases from each autosomal chromosome (nucleotide coordinates 20,100,000–20,200,000 in each of the 22 autosomes). Specifically, the above-mentioned read coverage values were used to calculate the ratio between mtDNA and nDNA read coverage. This ratio equals the estimated mtDNA copy numbers.

## Supporting Information

S1 Fig Differential expression pattern between studies. Normalized read count of RNA-seq samples from different published studies for L haplogroups (A-C) and non-L haplogroups (D-F). The Lappalainen dataset [26] shares most of the samples from the Pickrell [28] and Montgomery [29] datasets. (A) and (D) display the expression pattern of all mtDNA genes. ND1 and CO1 are shown as representative examples (B and E) and (C and F), respectively.
(TIF)

S2 Fig RNA read coverage of the mtDNA. Coverage of the mtDNA by the RNA reads for the long RNA dataset (A) and the tRNA dataset (B). X axis annotates the mtDNA positions, Y axis represents the different samples, and log10-transformed read count per position is represented by color (side bar).
(TIF)

S3 Fig Phylogenetic analysis of the extracted mtDNA sequences from RNA-seq and corresponding DNA sequences. A comparison between phylogenetic trees (NJ), based on 454 reconstructed mtDNA sequence of the long RNA dataset (A), and 402 mtDNA sequences extracted from the same individuals, which were part of the 1000 Genomes Project (B). Branch colors indicate the three macro haplogroups, L (red), M (blue) and N (purple). Arrows indicate the branches of the major haplogroups in the analysis, and bootstrap values of 1000 replicates for the specified haplogroup branches are indicated.
(TIF)

S4 Fig Comparison between expression analyses of mapping to chromosome M (rCRS) versus the mapping using the personalized mtDNA genomes. (A) Mapping of reads to rCRS (Chromosome M). (B) Mapping of reads against the personalized mtDNA genome of each sample. X axis–mtDNA genes, Y axis—normalized read count. Statistical significance: (*) p<3.7e-5; (**) p< 1e-6; (***) p< 1e-7.
(TIF)

S5 Fig Differentially expressed nDNA-encoded genes in L-haplogroup versus non-L haplogroup samples. Expression ratio of 2,380 differentially expressed nuclear genes in L versus non-L haplogroup samples. X axis represents the different genes, Y axis is the L/non-L ratio of the normalized read counts.
(TIF)

S1 Table mtDNA eQTL test of the long RNA dataset. Number of individuals per SNP are listed. Information indicated in columns E-H is available only for SNPs that passed the p-value cutoff after Bonferroni correction (3.7e^-5^) in the two tested groups.
(XLSX)

S2 Table mtDNA eQTL test of the tRNA dataset. Number of individuals per SNP are listed. Information indicated in columns E-H is available only for SNPs that passed the p-value cutoff after Bonferroni correction (3.47e^-5^) in the two tested groups.
(XLSX)

S3 Table List of nuclear genes that co-express with the mtDNA-encoded genes. (XLSX)

S4 Table mtDNA eQTL test of the Africans long RNA dataset. Number of individuals per SNP is listed. Information indicated in columns E-H is available only for SNPs that passed the p-value cutoff after Bonferroni correction (5.84e^-5^) in the two tested groups.
(XLSX)

S5 Table mtDNA eQTL test of the Caucasians long RNA dataset. Number of individuals per SNP is listed. Information indicated in columns E-H is available only for SNPs that passed the p-value cutoff after Bonferroni correction (4.22e^-5^) in the two tested groups.
(XLSX)

S6 Table mtDNA eQTL test of the Africans tRNA dataset. Number of individuals per SNP is listed. Information indicated in columns E-H is available only for SNPs that passed the p-value cutoff after Bonferroni correction (5.48e^-5^) in the two tested groups.
(XLSX)

S7 Table mtDNA eQTL test of the Caucasians tRNA dataset. Number of individuals per SNP is listed. Information indicated in columns E-H is available only for SNPs that passed the p-value cutoff after Bonferroni correction (3.95e^-5^) in the two tested groups.
(XLSX)

S8 Table Molecular function gene ontology (GO) annotation. (XLSX)

S9 Table nDNA eQTL test of the long RNA dataset. Information indicated in columns B-C is available only for SNPs that passed the p-value cutoff after Bonferroni correction (6.52e^-6^) in the two tested groups.
(XLSX)

S10 Table nDNA eQTL test of the tRNA dataset. Information indicated in columns B-C is available only for SNPs that passed the p-value cutoff after Bonferroni correction (6.11e^-6^) in the two tested groups.
(XLSX)
