# Supplementary figures and images for: Ancient Out-of-Africa Mitochondrial DNA Variants Associate with Distinct Mitochondrial Gene Expression Patterns
Source: PLoS Genet. 2016 Nov 3;12(11):e1006407. doi: 10.1371/journal.pgen.1006407 (PMC5094714; doi:10.1371/journal.pgen.1006407)

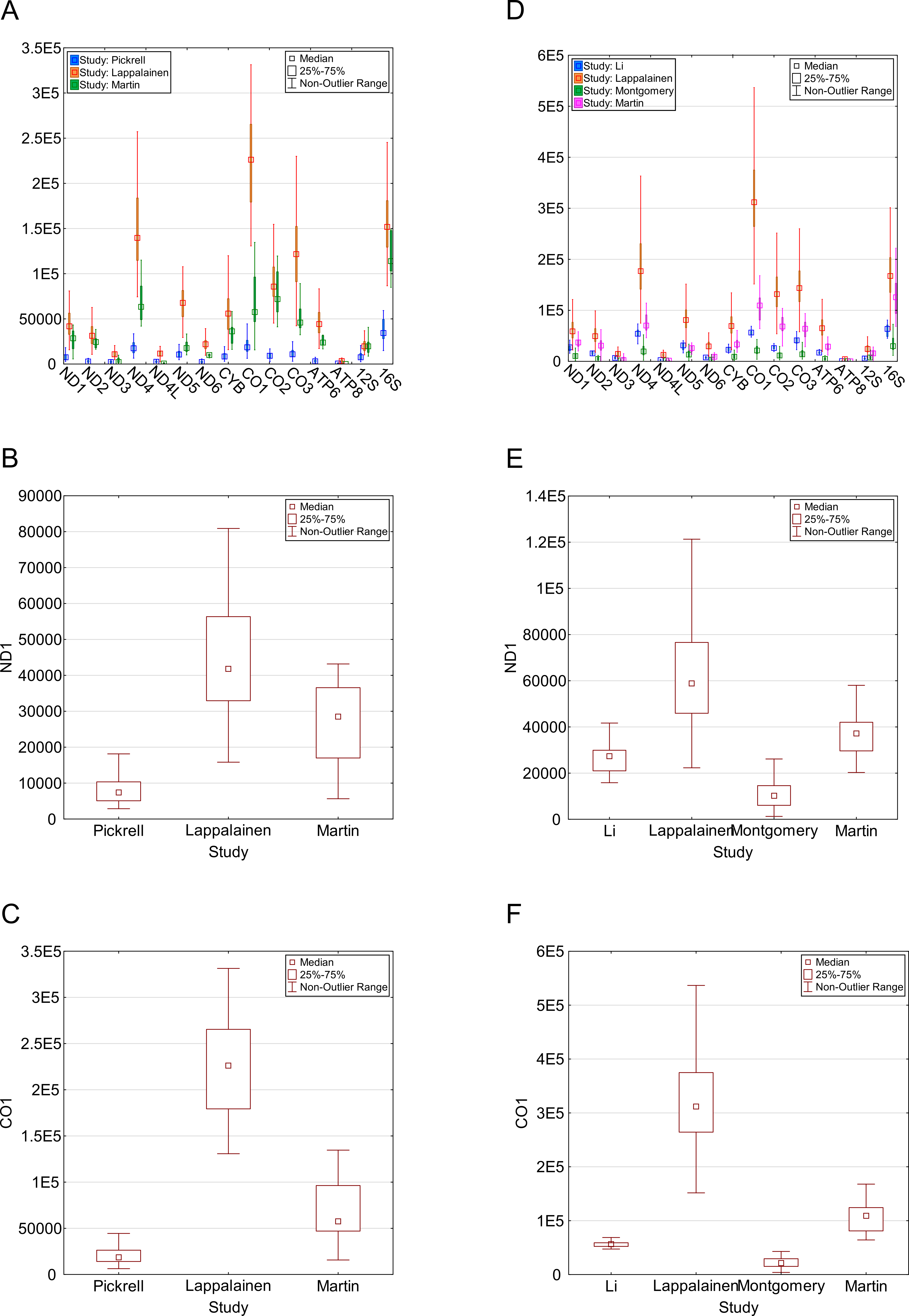

Supplement: S1 Fig — Normalized read count of RNA-seq samples from different published studies for L haplogroups (A-C) and non-L haplogroups (D-F). The Lappalainen dataset [26] shares most of the samples from the Pickrell [28] and Montgomery [29] datasets. (A) and (D) display the expression pattern of all mtDNA genes. ND1 and CO1 are shown as representative examples (B and E) and (C and F), respectively. (TIF) [file pgen.1006407.s001.tif]

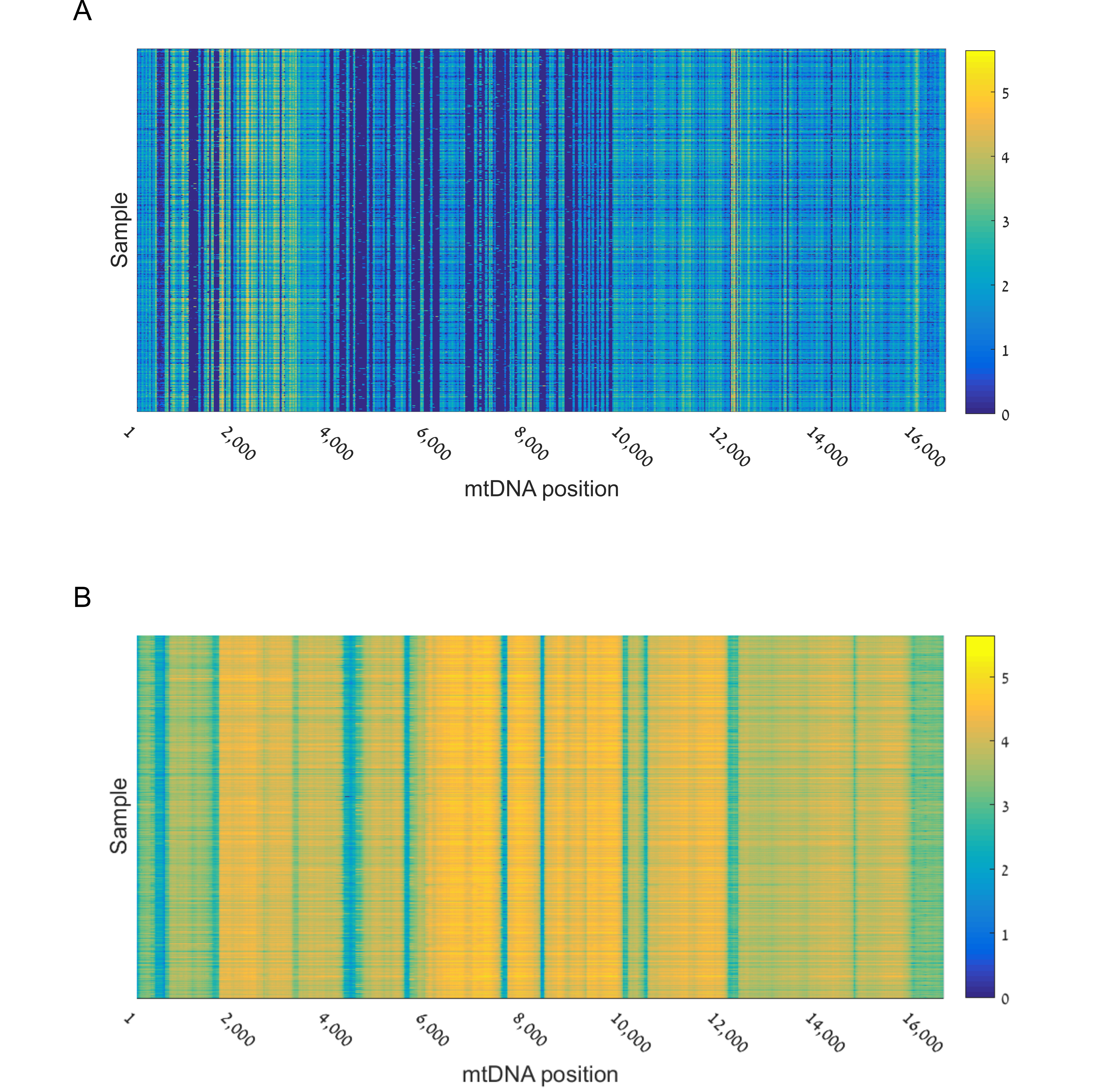

Supplement: S2 Fig — Coverage of the mtDNA by the RNA reads for the long RNA dataset (A) and the tRNA dataset (B). X axis annotates the mtDNA positions, Y axis represents the different samples, and log10-transformed read count per position is represented by color (side bar). (TIF) [file pgen.1006407.s002.tif]

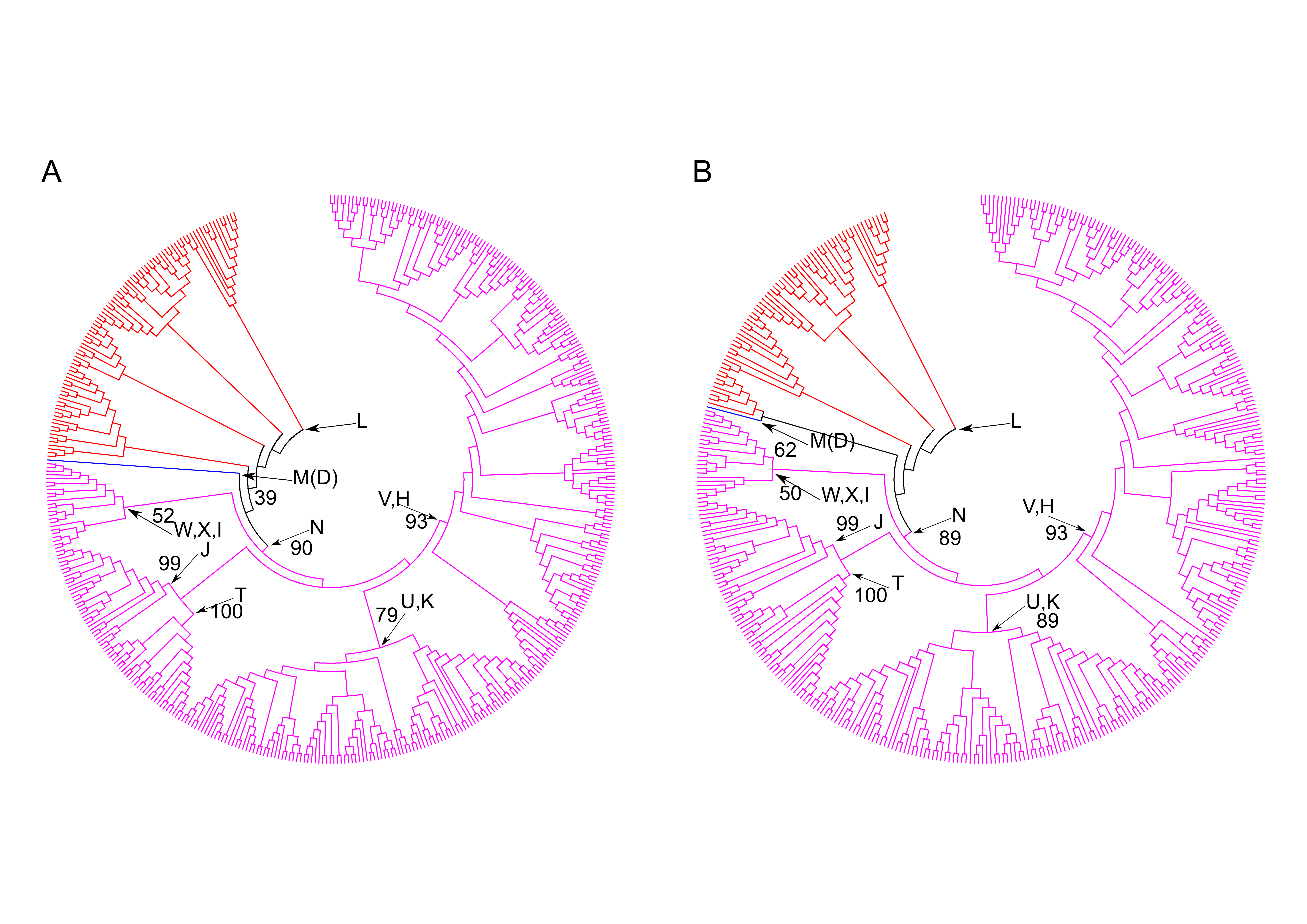

Supplement: S3 Fig — A comparison between phylogenetic trees (NJ), based on 454 reconstructed mtDNA sequence of the long RNA dataset (A), and 402 mtDNA sequences extracted from the same individuals, which were part of the 1000 Genomes Project (B). Branch colors indicate the three macro haplogroups, L (red), M (blue) and N (purple). Arrows indicate the branches of the major haplogroups in the analysis, and bootstrap values of 1000 replicates for the specified haplogroup branches are indicated. (TIF) [file pgen.1006407.s003.tif]

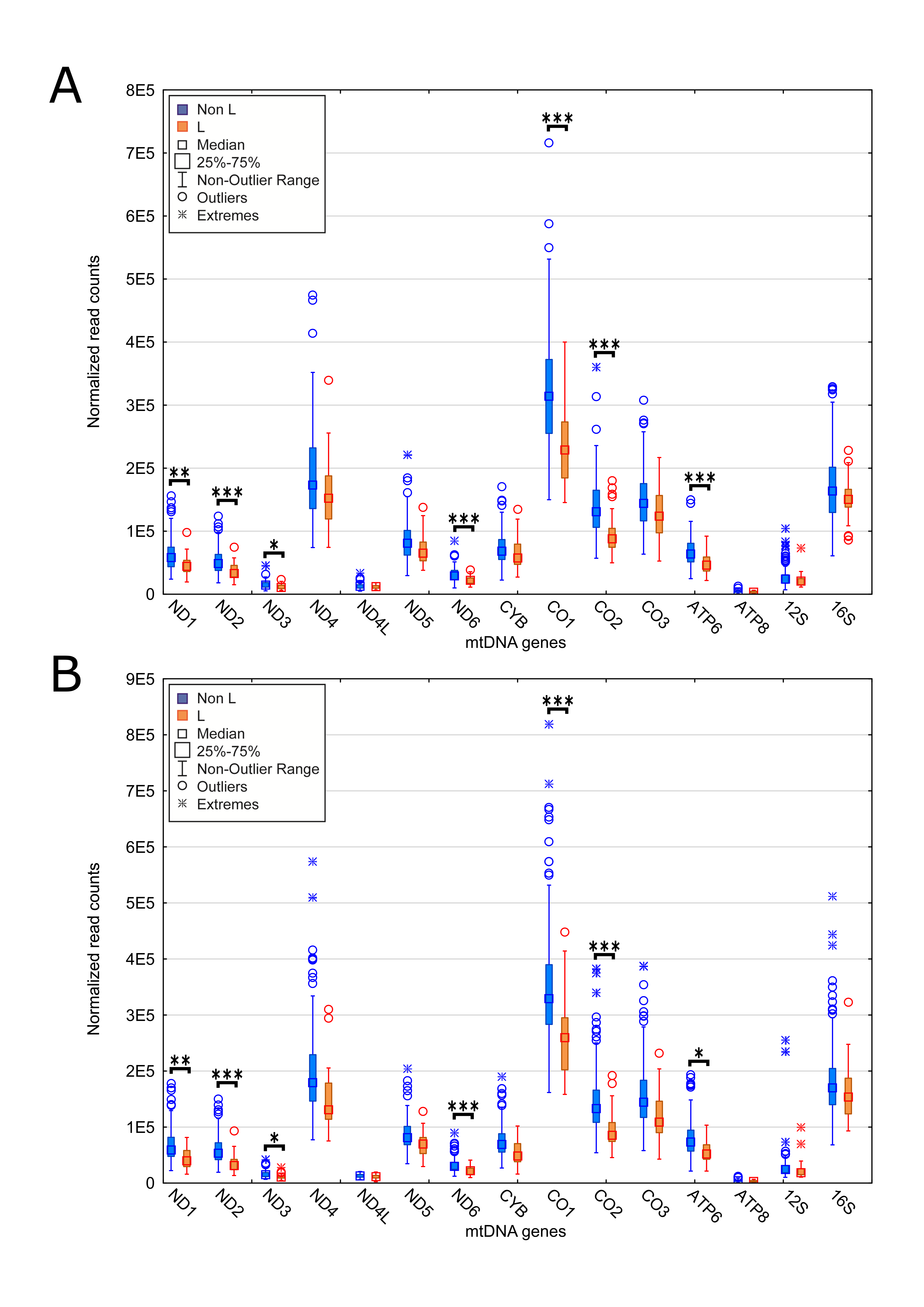

Supplement: S4 Fig — (A) Mapping of reads to rCRS (Chromosome M). (B) Mapping of reads against the personalized mtDNA genome of each sample. X axis–mtDNA genes, Y axis—normalized read count. Statistical significance: (*) p<3.7e-5; (**) p< 1e-6; (***) p< 1e-7. (TIF) [file pgen.1006407.s004.tif]

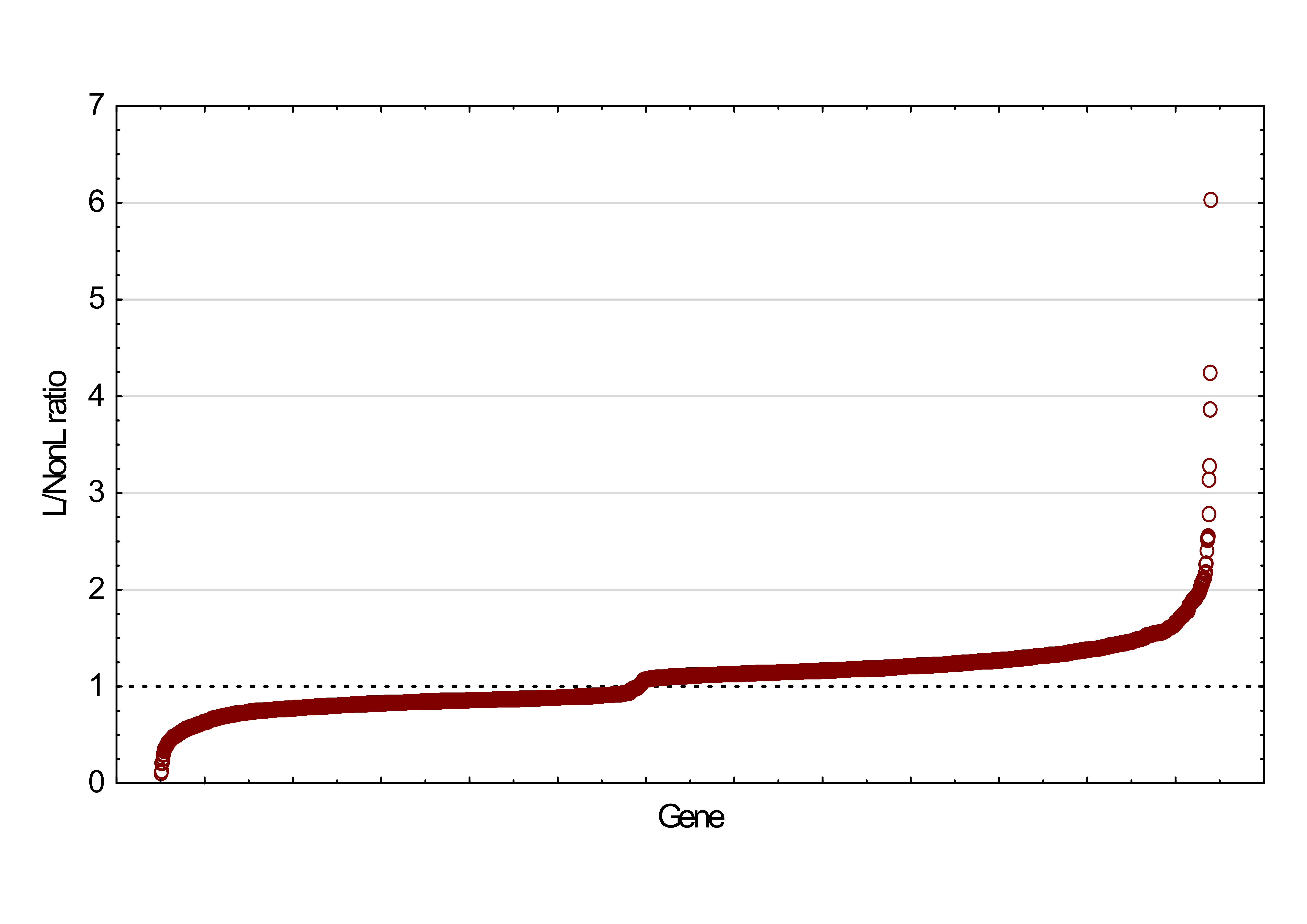

Supplement: S5 Fig — Expression ratio of 2,380 differentially expressed nuclear genes in L versus non-L haplogroup samples. X axis represents the different genes, Y axis is the L/non-L ratio of the normalized read counts. (TIF) [file pgen.1006407.s005.tif]
